# Letter to the Editor Re: Borschel M., et al. Comparison of Growth of Healthy Term Infants Fed Extensively Hydrolyzed Protein- and Amino Acid-Based Infant Formulas. *Nutrients* 2018, *10*, 289

**DOI:** 10.3390/nu11010185

**Published:** 2019-01-17

**Authors:** Bryan M. Harvey, Jane E. Langford

**Affiliations:** 1 Children’s Investigational Research Program, LLC (CHIRP)/Harvey Pediatrics, Rogers, AR 72758, USA; harveypeds@yahoo.com; 2 Global Medical Affairs, Nutricia Advanced Medical Nutrition, Schiphol Airport, 1118 BG Amsterdam, The Netherlands

**Keywords:** infant growth, amino acid-based formula, elemental formula, cow’s milk allergy, multiple food protein intolerance

We read with interest the recently published narrative review of seven growth studies in healthy infants fed extensively hydrolyzed protein-based formulas (eHF) and amino acid-based formulas (AAF) [1]. We do not agree with the points raised in this paper related to the lower growth patterns observed for some formulas, the analytical methodology used and the extrapolation of the findings from healthy infants to clinical practice. 

This review states that “differences in growth patterns were observed with some formulas supporting normative growth patterns during the first four months but others appearing to support markedly lower growth patterns”. We strongly refute this observation for AAF-F (Neocate® Infant) and AAF-I (Neo-Syn—commercialized as Neocate® Syneo) (study formula codes from [1]). Data we have collected for AAF-F and AAF-I show normal growth patterns when fed as a sole source of nutrition to healthy infants [2].

Borschel et al. [1] commented that in our paper [3], we did not present growth data compared to normal growth standards. Instead, we reported on the growth and tolerance outcomes for 115 healthy infants taking AAF-I with a synbiotic blend (test; Neo-Syn) compared to the commercially available AAF-F (control; Neocate Infant). The primary objective of our study was to establish whether the test formula promoted equivalent growth to a commercially available AAF. We presented comparisons of weight, length and head circumference for the two groups as ratios of these growth parameters between the two study groups and as weight gain and achieved length over the study period rather than as a comparison with growth standards. Although not reported in our paper [3], the growth (weight-, length- and head circumference-for-age) of both the test (AAF-I) and control (AAF-F) groups compared favourably to the WHO growth standards [4]. We found that infants in both AAF-I and AAF-F groups had growth that was comparable to healthy breastfed infants of the same age at each study visit, i.e., group mean *z*-scores for weight-, length- and head circumference-for-age were close to zero. These findings have been reviewed by the US Food and Drug Administration. 

Borschel et al. [1] extracted the growth outcomes from another study [5] in which AAF-F was fed as a control formula. The authors reported that infants fed AAF-F grew along the 30th to 37th centiles, similar to the growth rates found for the test group in their study (AAF-H). We note that for the control group on AAF-F, the mean (± SD) weight gain (g/day) for boys was 28.97 (± 4.99) and for girls was 25.37 (± 4.15), which is similar to normal weight gain reported for breastfed boys of 29.8 (± 5.8) and girls of 26.2 (± 5.6) [6]. In their narrative review, Borschel et al. [1] also extrapolated the growth findings for AAF-F from Corkins et al. [5] to infants fed AAF-I (the test group in our study [3]) and concluded that infants fed AAF-I would be expected to grow no better than those on AAF-F. The limitations of extrapolation are well-known but especially relevant in this case given that there are compositional differences between the formulations of AAF-I (Neo-Syn: 33% medium-chain triglycerides (MCT) and a synbiotic blend) and AAF-F [3] (Neo: 4% MCT and no synbiotic blend), and therefore it is particularly inappropriate to extrapolate the findings.

In terms of the methodology undertaken in Borschel et al. [1], ten formula codes are included and the probability of concluding a difference among formulas where none exists is high at approximately 90% assuming all possible pairwise comparisons. The issue of multiplicity is key given that the authors of the review recommend that eHFs and AAFs have to be chosen carefully in clinical practice due to their observations of differences in growth patterns. The review [1] states that “it was not a systematic review and may be subject to omission and/or bias.” The *t*-tests that were applied resulted in naive indirect comparisons: comparisons of the results of individual arms from different trials as if they were from the same randomised trial. This provides evidence equivalent to that of observational studies without any test or correction for confounding. Methods that perform adjusted indirect comparisons, like a network meta-analysis, generate results that are much more reliable, especially if the presence of heterogeneity and incoherence can be tested. This would have at least alerted the authors not to make misguided recommendations for the clinicians managing these infants. We are also concerned about the selective nature of the between-formula comparisons that were made. For example, the authors compare the mean weight gain per day (unadjusted comparisons) of AAF-F with AAF-G but make no other comparisons, such as AAF-G with AAF-H. 

Finally, one of the stated aims of the review [1] was that information on differences in growth performance in healthy infants may be of use to clinicians managing infants with medical conditions requiring these formulas. Whilst studies in healthy infants are informative, they do not answer the clinically relevant question of how well these formulas promote growth in the intended population. This is dependent not only on a formula’s nutritional composition but also on how well it is tolerated by infants for whom standard cow’s milk-based infant formula or breastmilk is clinically contraindicated. A systematic review of the literature concluded that children with food allergies have a higher risk of growth impairment [7]. The majority of studies over the last three decades that have established AAFs as effective in promoting growth in infants with food allergic disease and other gastrointestinal conditions have used Neocate®. An extensive evidence base in both observational [8,9,10,11,12,13,14] and randomised, controlled multicentre studies [15,16,17,18,19,20], in over 700 infants and children, have demonstrated that Neocate® and Neocate® Syneo (AAF-F and AAF-I) promote healthy growth and catch up growth in infants and children with food allergic disease.
